# Possible association between SIRT1 single nucleotide polymorphisms and predisposition to antisocial personality traits in Chinese adolescents

**DOI:** 10.1038/s41598-017-01208-2

**Published:** 2017-04-24

**Authors:** Hongjuan Chang, Qiuge Yan, Jie Tang, Juan Huang, Yanmei Zhang, Yuqiao Ma, Xiaozhou Ye, Lina Tang, Linguo Wu, Chunxia Wu, Yizhen Yu

**Affiliations:** 1 The Department of Child, Adolescence and Woman Health Care, School of Public Health, Tongji Medical College, Huazhong University of Science and Technology, Wuhan, China; 2 Guangzhou Medical University, Guangzhou, Guangdong China

## Abstract

Accumulating evidence suggests an association between the SIRT1 gene and human psychiatric disorders. The aim of the study was to investigate the association between SIRT1 and predisposition to antisocial personality traits (ASP) in Chinese adolescents. Participants consisted of 327 controls and 261 juvenile offenders who were diagnosed with predisposition to ASP according to the Personality Diagnostic Questionnaire. Four tag single nucleotide polymorphisms (tagSNPs) of SIRT1, namely rs12778366, rs7896005, rs10823112, and rs4746720, were genotyped. Association analysis between individual SNPs and ASP risk revealed the CC genotype of rs4746720 to be significantly associated with reduced risk of ASP (OR = 0.51, 95% CI = 0.33–0.77, adjusted *P* = 0.007). Haplotype analysis showed the TAAC haplotype was associated with reduced susceptibility to ASP (OR = 0.72, 95% CI = 0.57–0.91, *P* = 0.005). Moreover, rs4746720 variants were found to not only have a direct impact on ASP susceptibility but also modulate the effect of alcohol consumption (Y = 0.022X + 0.431 vs. Y = −0.066X + 0.387). The present study is the first to report a significant association between SIRT1 polymorphisms and ASP in adolescents. This finding is expected to aid in the development of effective interventions for this socially and personally costly disorder.

## Introduction

Antisocial personality traits (ASP) represent a serious psychiatric condition that poses a significant burden on the affected individuals and society^1^, and lacks an effective treatment^1, 2^. ASP is defined as a “pervasive pattern of disregard for, and violation of, the rights of others”. This pattern of behavior typically starts in childhood or adolescence and persists through adulthood^3^. ASP and major psychiatric disorders, such as mood swings, anxiety, and substance abuse, are common and present high comorbidity^4, 5^. The disorder has been well studied in community samples and is estimated to affect 4.4% of the general population^6^. It should be noted that ASP is particularly common in prison settings, with a worldwide prevalence of about 50% among male prisoners^7, 8^.

It is commonly accepted that genetics plays a substantial role in the etiology of psychopathic disorders, particularly in ASP^9–11^. Familial and twin registry studies indicate that ASP is highly heritable, with genetic factors accounting for 40–69% of the variation in diagnoses^12–14^. One neurochemical system that has been implicated, both theoretically and empirically, in ASP and psychopathy is the serotonin system (which consists of the 5-HTTLPR polymorphism in SLC6A4 and the genes HTR1B, MAOA, and MAOB)^15–18^. Recently, a genome-wide association study (GWAS) revealed that rs4714329 reached genome-wide significance (OR = 1.59, 95% CI = 1.37–1.85, *P* = 1.6 × 10^−9^)^19^. Genome-wide studies have confirmed only a minor contribution of single genetic polymorphisms to the incidence of ASP; thus, they are difficult to replicate. In summary, the etiology of ASP remains complex and poorly understood.

The SIRT family of nicotinamide adenine dinucleotide (NAD)-dependent deacetylases comprises seven members (SIRT1–7). These are emerging as having an important role in the regulation of metabolism, aging, oxidative stress, and circadian rhythms, and as modulators of synaptic plasticity and memory formation^20–23^. SIRT1, which localizes mainly to the nucleus, regulates gene transcription and DNA repair^24^. As such, SIRT1 plays an important functional role in numerous biological processes. One of these is the circadian clock system^25^, whereby SIRT1 associates and is recruited to the BMAL1 chromatin complex at circadian promoters^26^. Importantly, many studies evoke the involvement of circadian rhythm disruption in several psychiatric disorders such as, schizophrenia, anxiety disorders, bipolar disorders, and aggressive behaviors, which is the main characteristic of ASP^27–29^. Furthermore, a GWAS has identified various SIRT1 polymorphisms that confer susceptibility to major depressive disorders^30^. Notably, evidence has emerged showing that the SIRT1 gene is associated with anxiety disorders in animals^31^. Based on the above, we considered the SIRT1 gene to be a good candidate for ASP. At present, it is not known whether the SIRT1 gene is related to ASP.

Complex behavioral patterns such as those characteristic of ASP result from a combination of genetic and environmental factors, with a possible role for environmental experiences such as maltreatment during childhood^32^. Strikingly, previous studies have underscored the importance of the close interplay between genetic and environmental factors in the etiology of ASP. It has been shown that a functional polymorphism in the MAOA gene moderates the impact of childhood maltreatment on the development of ASP^33^. Biologically, childhood maltreatment has been shown to promote changes in brain structure, atypical development of the hypothalamic-pituitary-adrenal axis, elevated neurotransmitter levels, and gene methylation^34–36^. Based on the existing research on ASP, we hypothesize that: (1) The SIRT1 gene may be linked to ASP. (2) The SIRT1 gene may interact with environmental factors in determining the risk of ASP.

The aim of the present case-control study was to identify common genetic variants underlying variation and interplay between genetic and environmental factors in a population of Chinese adolescents with ASP. In particular, we attempted to obtain evidence for an association between ASP and SIRT1 in this population.

## Results

### Characteristics of the study subjects

Table 1 summarizes the characteristics of the cases and controls included in the study. The mean ages for the ASP and control groups were similar (19.79 ± 2.66 vs. 19.51 ± 2.44 years). Frequency of alcohol consumption, tobacco smoking (on the basis of which subjects were classified into the often, sometimes, and never groups, as shown in Table 1), and history of childhood maltreatment differed significantly between case and control groups (*P* < 0.001) (Table 1). When controlling for other environment variables (For example, alcohol consumption, network usage, emotional abuse, physical abuse, sex abuse emotional neglect and physical neglect were controlled, when calculate independent effect of tobacco smoking), only tobacco smoking (OR = 19.50, 95% CI = 10.87–34.99, *P* < 0.001) and sex abuse (OR = 21.35, 95% CI = 5.73–79.58, *P* < 0.001) were predictors of ASP, according to logistic regression.

**Table 1:** Characteristics of the study sample.

Variables	Cases (*n* = 261)	Controls (*n* = 327)	χ^2^/*t*	*P*	OR (95% CI)	*P*
**Age (in years)**	19.79 ± 2.66	19.51 ± 2.44	1.27	0.204	0.92 (0.79–1.06)	0.24
**Tobacco smoking**			**400.21**	**<0.001**	**19.50 (10.87–34.99)**	**<0.001**
never	15	277				
sometimes	51	41				
Often	192	9				
**Alcohol consumption**			**107.73**	**<0.001**	1.17 (0.53–2.11)	0.688
never	41	105				
sometimes	138	217				
Often	82	5				
**Network usage**			**51.50**	**<0.001**	1.11 (0.59–2.11)	0.743
never	22	15				
sometimes	46	151				
Often	188	161				
**Emotional abuse**			153.99	**<0.001**	3.66 (0.99–13.63)	0.053
none to moderate	121	300				
moderate to extreme	129	18				
**Physical abuse**			145.97	**<0.001**	**4.39 (0.98–21.21)**	**0.066**
none to moderate	136	302				
moderate to extreme	117	11				
**Sex abuse**			141.27	**<0.001**	**21.35 (5.73–79.58)**	**<0.001**
none to moderate	141	296				
moderate to extreme	114	9				
**Emotional neglect**					**1.80 (0.67–4.83)**	**0.245**
none to moderate	113	265	93.12	**<0.001**		
moderate to extreme	136	51				
**Physical neglect**			103.13	**<0.001**	**1.49 (0.56–3.99)**	**0.423**
none to moderate	110	270				
moderate to extreme	146	54				

Data calculated by two independent sample *t-*tests and logistic regression and other variables were controlled.

Positive results are in bold. Variable may not add up to the total number due to missing values.

### Characteristics of tagSNPs

Four single nucleotide polymorphisms (SNPs) were successfully genotyped in a total of 588 subjects. SIRT1 was mapped to chromosome 10q21.3, and was approximately 33.7 kb in length. The distribution of genotypes did not deviate from the Hardy-Weinberg equilibrium in any of the groups or in the overall sample (*P* > 0.05). The general characteristics of tagSNPs are shown in Table 2. Minor allele frequencies (MAFs) of all four tagSNPs in our controls were similar to MAFs for CHBS_1000 g.

**Table 2:** Characteristics of the four SNPs included in the current study.

IDs	Location	Reference/Variant allele	W/H/V frequency (cases)	W/H/V frequency (controls)	MAF in cases	MAF in controls	MAF in CHBS_1000 g	*P* for HWE
rs12778366	5′FLANKING	T/C	193/65/3	251/67/9	0.136	0.130	0.132	0.089
rs7896005	intron2	A/G	178/77/6	243/77/7	0.171	0.139	0.157	0.757
rs10823112	intron6	A/G	133/106/22	191/113/23	0.287	0.243	0.325	0.270
rs4746720	3′UTR	T/C	88/134/39	92/151/84	0.406	0.488	0.386	0.170

Abbreviations: W, wild type homozygote; H, heterozygote; V, variant homozygote.

MAF, minor allele frequency; CHB, Han Chinese in Beijing, China; HWE, Hardy-Weinberg equilibrium.

### Association analysis between individual SNPs and ASP risk

Allele and genotype distributions of the four tagSNPs in cases and controls are shown in Table 3. Based on genetic model association analysis, only rs4746720 showed statistical significance. Compared to the CT/TT genotype, the CC genotype was associated with reduced AB risk (OR = 0.51, 95% CI = 0.33–0.77, adjusted *P* = 0.007) in a recessive model. C allele frequency of rs4746720 in patients with ASP was significantly lower than in the control group in an additive model (OR = 0.72, CI = 0.57–0.91, adjusted *P* = 0.024). Taken together, our data suggest that the SIRT1 tagSNP rs4746720 is associated with ASP risk, and that individuals carrying the C allele exhibit significantly reduced ASP susceptibility. This confirms hypothesis (1). It should be noted that we did not detect any association between rs12778366, rs7896005, or rs10823112 and the risk of ASP in allelic or genotypic analyses.

**Table 3:** Association analysis between individual SNPs and ASP risk.

SNP	Variables	Cases (%)	Controls (%)	OR (95% CI)	*P*	FDR_BH adjusted
rs12778366	TT	193 (73.9)	251 (76.8)	1		
	TC	65 (24.9)	67 (20.5)	1.26 (0.86–1.86)	0.242	
	CC	3 (1.1)	9 (2.8)	0.43 (0.12–1.62)	0.215	0.426
	Dom.			1.16 (0.80–1.70)	0.431	0.431
	Rec.			0.41 (0.11–1.53)	0.186	0.371
	Add.			1.05 (0.75–1.47)	0.764	0.764
rs7896005	AA	178 (68.2)	243 (74.3)	1		
	AG	77 (29.5)	77 (23.5)	1.37 (0.94–1.98)	0.099	
	GG	6 (2.3)	7 (2.1)	1.17 (0.39–3.54)	0.781	0.781
	Dom.			1.35 (0.94–1.93)	0.103	0.193
	Rec.			1.08 (0.36–3.24)	0.897	0.897
	Add.			1.28 (0.93–1.76)	0.137	0.182
rs10823112	AA	133 (51.0)	191 (58.4)	1		
	AG	106 (40.6)	113 (34.6)	1.35 (0.95–1.90)	0.091	
	GG	22 (8.4)	23 (7.0)	1.37 (0.74–2.57)	0.953	0.426
	Dom.			1.35 (0.97–1.88)	0.071	0.193
	Rec.			1.22 (0.66–2.24)	0.528	0.704
	Add.			1.25 (0.96–1.61)	0.094	0.182
rs4746720	TT	88 (33.7)	92 (28.1)	1		
	CT	134 (51.3)	151 (46.2)	0.93 (0.64–1.35)	0.694	
	CC	39 (14.9)	84 (25.7)	**0.49 (0.30–0.78)**	**0.003**	**0.012**
	Dom.			0.77 (0.54–1.09)	0.145	0.193
	Rec.			**0.51 (0.33–0.77)**	**0.002**	**0.007**
	Add.			**0.72 (0.57–0.91)**	**0.006**	**0.024**

Abbreviations: Dom, dominant model; Rec, recessive model; Add, additive model; OR, odds ratio; 95% CI, 95% confidence interval; FDR_BH, false discovery rate-Benjamini and Hochberg.

Positive results are in bold.

### Association between SIRT1 tagSNPs haplotypes and ASP risk

**Figure 1:**
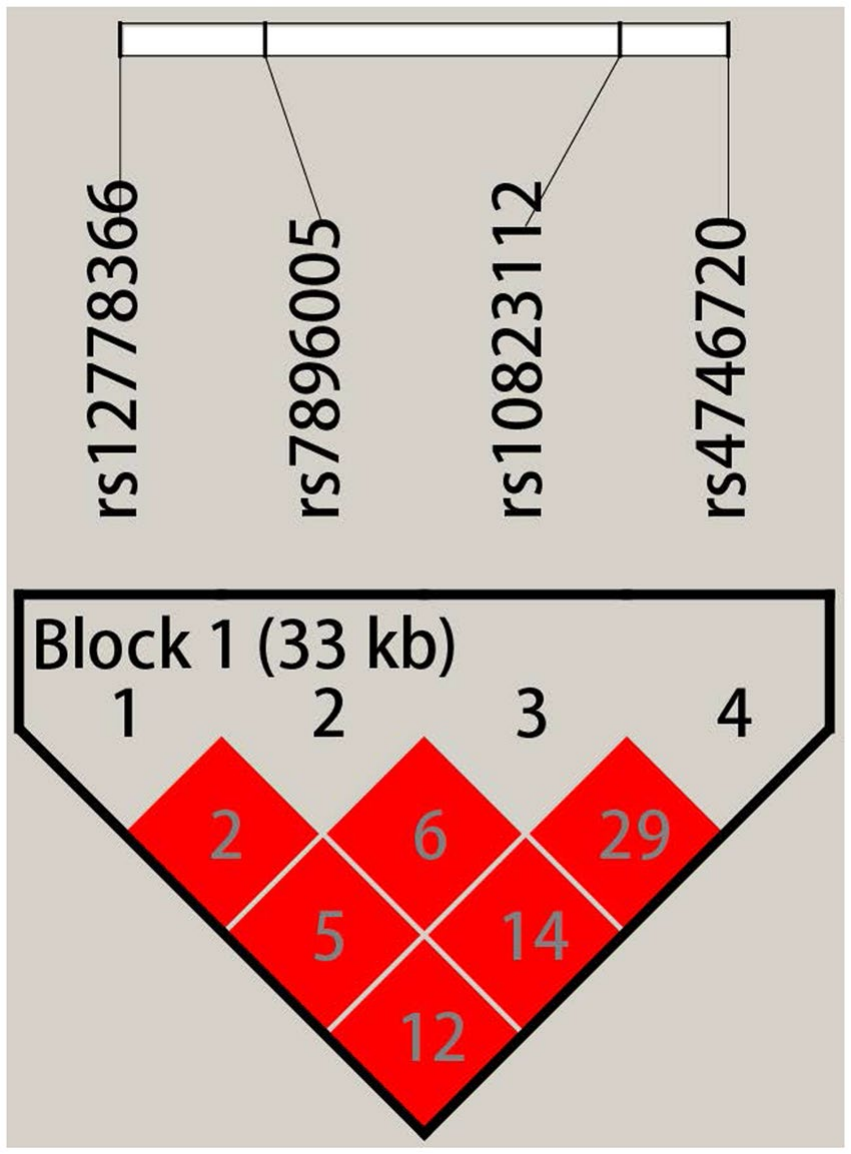
Four tagSNPs haplotypic block analysis.

As shown in Fig. 1, all four tagSNPs were located in one haplotypic block. Therefore, we further compared the haplotype frequencies of the four tagSNPs between the ASP group and controls. Four common haplotypes (frequency >3%) derived from the four tagSNPs accounted for almost 100% of haplotype variations. Among the four common haplotypes, only rs12778366T-rs7896005A-rs10823112A-rs4746720C was associated with a reduced ASP risk (OR = 0.72, 95% CI = 0.57–0.91 *P* = 0.005, Table 4).

**Table 4:** Association between SIRT1 tagSNP haplotypes and the risk of ASP.

Haplotype	Cases (%)	Controls (%)	OR	95% CI	*P*
TAGT	150 (0.29)	79 (0.24)	1.26	0.97–1.63	0.087
CAAT	71 (0.14)	39 (0.12)	1.05	0.75–1.48	0.761
**TAAC**	**212 (0.41)**	**151 (0.46)**	**0.72**	**0.57–0.91**	**0.005**
TGAT	89 (0.17)	57 (0.18)	1.27	0.93–1.75	0.139

Positive results are in bold.

### Association of pairwise interactions with ASP risk by logistic and moderate analysis

**Figure 2:**
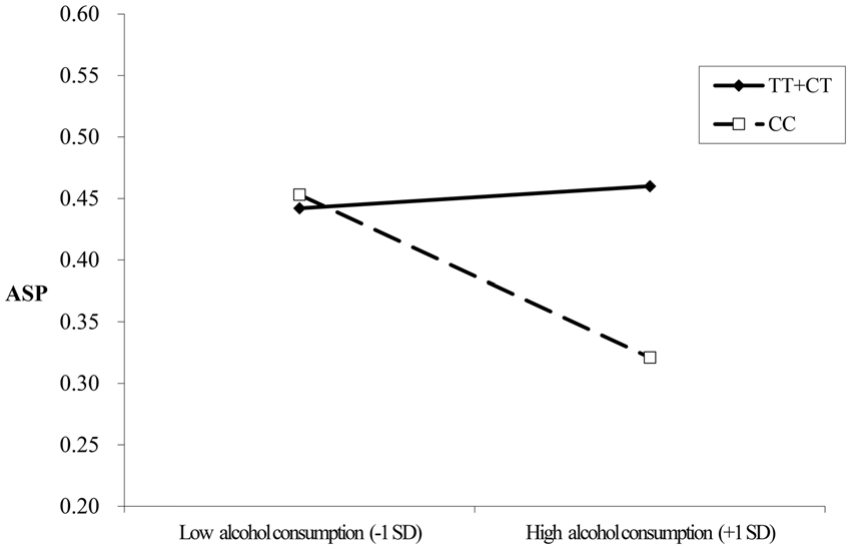
Relationship between rs4746720 and alcohol consumption, and ASP risk.

Compared to the CT/TT genotype in the often-tobacco smoking group, the CC genotype was associated with reduced ASP risk (OR = 0.21, 95% CI = 0.05–0.84) by univariate logistic regression; however, the interaction was not significant according to multivariate logistic regression. Furthermore, the CC genotype exhibited reduced ASP risk compared to the CT/TT genotype in the sometimes- (OR = 0.44, 95% CI = 0.25–0.77) and often-alcohol consumption groups (OR = 0.09, 95% CI = 0.01–0.62) (Table 5). The rs4746720 CC genotype exhibited reduced ASP risk compared to the CT/TT genotype in the subgroup defined by maltreatment history in childhood. Childhood experiences of emotional, physical, and sexual abuse, as well as physical and emotional neglect were all strong predictors of ASP (*P* < 0.001). The interaction between rs4746720 genotype and alcohol consumption was statistically significant (OR = 0.43, 95% CI = 0.20–0.94, *P* = 0.034). However, no other statistically significant gene-environmental interactions were detected (Table 5). Furthermore, rs4746720 variants were found to not only have a direct impact on ASP susceptibility but to also modulate the effects of alcohol consumption (Y = 0.022X + 0.431 vs. Y = −0.066X + 0.387) (Table 6, Fig. 2), thus confirming also hypothesis (2).

**Table 5:** Pairwise interactions between genetic variants of rs4746720.

Variables	OR (95%CI)^a^		OR (95%CI)^b^	OR (95%CI)^b^	OR (95%CI)^b^
	CT + TT	CC	Variables	rs4746720	Interaction
**Tobacco smoking**			23.57 (14.22–39.07) ***	1.64 (0.24–10.95)	0.54 (0.22–1.37)
never	Ref.	0.71 (0.20–2.65)			
sometimes	Ref.	0.66 (0.23–1.90)			
Often	Ref.	**0.21 (0.05–0.84)**			
**Alcohol consumption**			4.69 (3.21–6.85)***	2.64 (0.56–12.53)	0.43 (0.20–0.94)*
never	Ref.	1.15 (0.49–2.69)			
sometimes	Ref.	**0.44 (0.25–0.77)**			
Often	Ref.	**0.09 (0.01–0.62)**			
**Network usage**			1.81 (1.33–2.46)***	0.39 (0.04–3.76)	1.07 (0.46–2.47)
never	Ref.	0.40 (0.24–0.68)			
sometimes	Ref.	0.78 (0.33–1.84)			
Often	Ref.	**0.40 (0.24–0.68)**			
**Emotional abuse**			15.38 (8.54–27.70)***	0.48(0.27–0.84)*	2.02 (0.48–8.53)
none to moderate	Ref.	**0.48 (0.27–0.84)**			
moderate to extreme	Ref.	0.97 (0.26–3.65)			
**Physical abuse**			21.15 (10.31–43.37)***	0.49 (0.28–0.83)**	1.68 (0.31–9.21)
none to moderate	Ref.	**0.49 (0.28–0.84)**			
moderate to extreme	Ref.	0.81 (0.16–4.10)			
**Sex abuse**			24.48 (11.01–54.43)***	0.45 (0.26–0.76)**	1.47 (0.26–8.34)
none to moderate	Ref.	**0.32 (0.14–0.73)**			
moderate to extreme	Ref.	0.52 (0.21–1.28)			
**Emotional neglect**			7.02 (4.48–10.98)***	0.66 (0.38–1.14)	0.55 (0.21–1.44)
none to moderate	Ref.	0.66 (0.38–1.14)			
moderate to extreme	Ref.	**0.37 (0.17–0.80)**			
**Physical neglect**			7.93 (5.05–12.45)***	0.55 (0.30–1.00)	0.58 (0.23–1.47)
none to moderate	Ref.	**0.55 (0.30–0.99)**			
moderate to extreme	Ref.	**0.32 (0.16–0.65)**			

^a^Data calculated by univariate logistic regression; ^b^Data calculated by multivariate logistic regression (Y = a0 + a1 X1 + a2 X2 + a1a2 X1 * X2); Positive results are in bold. *P < 0.05 (two-tailed), **P < 0.01 (two-tailed), ***P < 0.001 (two-tailed).

**Table 6:** Hierarchical regression analysis of the effect of rs4746720 polymorphisms on the relationship between alcohol consumption and ASP.

Variables	Step1 (β)	Step2 (β)	Step3 (β)	Step4 (β)
Constant	0.439***	0.439***	0.451***	0.451***
Age (in years)	−0.003	−0.003	−0.002	−0.002
Tobacco smoking	0.414***	0.416***	0.413***	0.412***
Network usage	−0.008	−0.007	−0.005	−0.006
Alcohol consumption		−0.005	−0.005	0.009
rs4746720 (Recessive)			−0.058*	−0.064*
rs4746720 (Recessive) × Alcohol consumption				−0.075**
ΔR^2^		0.000	0.002*	0.003**

*P < 0.05 (two-tailed), **P < 0.01 (two-tailed), ***P < 0.001 (two-tailed).

Positive results are in bold.

## Discussion

In the present case-control study, we investigated whether four tagSNPs in SIRT1 were associated with ASP risk in Chinese adolescents. Our results suggest that a genetic variant of rs4746720, namely the CC genotype, is significantly associated with a reduced risk of ASP. Furthermore, a significantly lower frequency of the rs12778366T-rs7896005A-rs10823112A-rs4746720C haplotype was observed in ASP cases compared to controls. Genetic background plays an important role in the development of ASP, a phenomenon that has been extensively investigated in recent years. SIRT1 is unlikely to be the only gene that modulates ASP risk. The role of other genes, most notably MAOA, in determining susceptibility to ASP remains to be elucidated^18, 37^. Compelling evidence suggests that numerous genes are involved in determining an individual’s predisposition to psychiatric disorders^38, 39^. Therefore, the combined role of multiple genes should be considered. In recent years, GWASs have identified multiple genetic loci associated with ASP^40, 41^. However, genetic information is merely a proxy for the underlying structure and function of biological systems; these can only be understood by determining the specific function of a gene.

Rs4746720 is a functional polymorphism located in the 3′ UTR region of SIRT1. We used haploreg software to predict the function of rs4746720, however there is no specific evidence supporting any functional or structural alterations to SIRT1 caused by this variant (http://archive.broadinstitute.org/mammals/haploreg/haploreg.php; http://www.regulomedb.org/). Nevertheless, the strong linkage between rs4746720 and rs75818614 (R^2^ = 0.86), and a likely effect of rs75818614 on the binding of transcription factors as deduced from ChIP-Seq experiments, suggests a possible function of rs474672042^42^. Moreover, it has been reported that, among healthy Han Chinese, carriers of the C allele variant of rs4746720 have a higher risk of natural aging than non-carriers^43^. Recently, preclinical studies have suggested that SIRT1 signaling influences the dopaminergic system^44, 45^ and is associated with defects in synaptic plasticity in the hippocampus^22, 46^. SIRT1 variations associated with ASP may exert biological effects by disrupting circadian rhythms and the dopaminergic system, thus leading to the development of ASP.

In line with the results of numerous previous studies, our findings highlight the significant role of childhood maltreatment in ASP risk. We suggest that the environment plays a major role in determining susceptibility to ASP; as a result, the effect of rs4746720 is relatively negligible. Additionally, we studied the effect of gene-environment interactions; however, the genetic variants examined in this study did not show strong effects when linked to certain environmental factors. It should be noted that epigenetic regulation may be at least partially mediated by the environment, resulting in lasting phenotypic effects^47^. Therefore, further investigation of the role of epigenetic regulation is necessary.

ASP often co-exists with alcohol-related disorders^48–50^. The highest estimates of ASP (over 70%) have been reported in clinically ascertained populations of males with substance use disorders^51^. Based on the findings herein, genotype and environment have a “fan-shaped” interaction. Its key characteristic is a modest difference in the level of the outcome variable as a function of genetic liability when individuals exhibit a “virtuous” conduct. In other words, the role of specific genes is minimal under such circumstances. More specifically, the genotype may act as a barrier when ASP co-exists with alcohol-related disorders, rendering the relationship between these two weak or non-existent, and raising questions of ASP comorbidity^52^. High scores of impulsivity/disinhibition traits are associated with alcohol-related problems, because individuals with high impulsivity scores are more likely to be diagnosed as alcoholics^53^. Therefore, the SIRT1 variant described in this study seems associated with the impulsivity antisocial trait rather than lack of empathy. Further research should be performed to allow for better grouping of antisocial personality traits. In addition, the researchers speculate that the genotype of SIRT1 may be associated with certain metabolic enzymes, which can affect individual alcohol consumption. But there is no literature reports. Further research should to explore the SIRT1 gene function when ASP co-exists with alcohol-related disorders. At the same time, mechanisms underlying the relationship between genotype and ASP remain relatively unexplored. As research on the behavioral genetics of ASP expands beyond studies of heritability, it becomes increasingly clear that understanding the role of genes and behavior requires the characterization of probabilistic and interdependent influences of genetic predispositions and comorbidity risk factors.

The main strengths of this paper are represented by the target group and the significant results obtained after correcting for multiple comparisons. The present tagSNPs contain almost all the information required for a genetic model. There are, nevertheless, also several limitations to this study, some of which are related to its methodology. First, the study was based on the use of self-report questionnaires; as such, the participants were not evaluated by a mental health professional to diagnose ASP. All participants in our sample were men; therefore, our findings may not be extrapolated to women. Third, experimental data from cell line or animal studies did not reveal any SNP function. Finally, sample size was relatively small; consequently, the results of this study should be confirmed by analyzing a larger sample. Despite these limitations, our study contributes significantly to the understanding of the role of SIRT1 tagSNPs on ASP risk in adolescence by demonstrating the importance of individuals’ genetic make-up; to our knowledge, this has not been identified in previous studies.

In summary, to our knowledge, no previous study has investigated whether SIRT1 genetic polymorphisms determined the onset of ASP in adolescents, or identified the underlying mechanisms. The present results provide preliminary evidence that SIRT1 polymorphisms may contribute to the risk of ASP. These findings may enable the elucidation of the genetic basis determining predisposition to ASP.

## Materials and Methods

### Participants

A total of 261 male juvenile offenders (under the age of 25 years) from youth correctional facilities of Hubei province, who were diagnosed with ASP according to Personality Diagnostic Questionnaire-4+ (PDQ-4+) criteria, were selected for our study. Juvenile offenders were excluded if they had severe mental retardation or a major physical illness. Finally, 327 volunteers recruited from universities and high schools, matched to the cases in terms of age, gender, place of birth, and ethnicity, were included in the control group. All participants were male and native of Hubei province. This study was reviewed and approved by the ethics committee of Tongji Medical College, Huazhong University of Science and Technology. The study was performed according to the approved ethical guidelines. Informed written consent was obtained from each participant prior to enrollment.

## Instruments

### Sociodemographic variables

Data regarding sociodemographic variables, such as age, place of birth, alcohol consumption, tobacco smoking, and social networks were obtained.

### Diagnostic assessment

The PDQ-4+ was used to evaluate ASP of juvenile delinquents and healthy individuals. PDQ-4+ is a self-report questionnaire composed of 107 true/false items. To date, the PDQ-4+ has been shown to have good reliability and validity, and has been widely used in clinical practice^54, 55^. A total score of 30 or more indicates that the subject has a personality disturbance. Cut-off scores for each subscale are 3–5^56^. Corresponding alpha coefficients were 0.85 in the current study. Scores higher than 5 indicated a disposition to ASP. This subscale included 8 items, the last of which contained 15 sub-items. The participants received a score of 1 if more than 3 sub-items were chosen for the last item.

### History of childhood maltreatment

Maltreatment history was evaluated using the Childhood Trauma Questionnaire^57^, a 28-item self-report instrument, developed by Bernstein, that evaluates emotional, physical, and sexual abuse, as well as physical and emotional neglect during childhood. Responses range from 1 (never true) to 5 (very true). Internal consistency in our study was high for the sample (Cronbach’s α = 0.75).

## Materials

### Gene selection

Tag SNPs were identified from the Han Chinese data set of the International HapMap Consortium (http://hapmap.ncbi.nlm.nih.gov/). First, the selected gene region was extended by 2000 base pairs both upstream and downstream in the genome. Haploview 4.2 software was then used to run the tagger program with the parameters set to a minor allele frequency of at least 0.1 in the Chinese Han population and a pairwise r^2^ of at least 0.8. Four tagSNPs of SIRT1 were selected: rs12778366, rs7896005, rs10823112, and rs4746720.

### Genotyping techniques

Genomic DNA was isolated from buccal epithelial cells using standard methods.

All genotypes of interest were tested for Hardy-Weinberg equilibrium using a goodness-of-fit χ^2^-test. SNP genotyping was performed using a custom-design 2 × 48-Plex SNPscan^TM^ kit (Genesky Biotechnologies Inc., Shanghai, China) based on double ligation and multiplex fluorescence PCR. To validate the genotyping accuracy of the kit, a 5% random sample of cases and controls was genotyped twice for all SNPs by different analysts. Specifically, we included 22 pairs of blind duplicates, and concordance rate was more than 98%.

### Statistical and bioinformatics analyses

All statistical analyses were performed using SPSS 18 software (SPSS Inc., Chicago, IL, USA). Means and standard deviations were computed for continuous variables. Counting data was analyzed using a χ^2^- test. Univariate or multivariate logistic regression analysis was performed to calculate the OR and 95% CI for the factors related to ASP. Multiple linear regression models were generated by entering the covariates first, followed by the predictors, and then the interaction term. Changes in R^2^ value between each step and the *P* values associated with the R^2^ change were noted. SHEsis software (http://shesisplus.bio-x.cn/SHEsis.html) was used to test a possible association of statistically inferred haplotypes with ASP. All presented *P* values are two-sided, and *P* < 0.05 was considered to represent statistical significance. The false discovery rate adjustment was applied for multiple comparisons.
